# Perivascular Adipocytes’ Adipogenesis Is Defined by Their Anatomical Location in the Descending Thoracic Aorta

**DOI:** 10.3390/cells14080579

**Published:** 2025-04-11

**Authors:** G. Andres Contreras, C. Javier Rendon, Alyssa Shadowens, Miguel Chirivi, David Salcedo-Tacuma, D. Adam Lauver, Stephanie W. Watts

**Affiliations:** 1 Department of Large Animal Clinical Sciences, Michigan State University, East Lansing, MI 48824, USA; rendonj1@msu.edu (C.J.R.); shadowe1@msu.edu (A.S.); chirivim@msu.edu (M.C.); 2 Department of Biochemistry and Molecular Medicine, West Virginia University School of Medicine, 4 Medical Center Dr., Morgantown, WV 26506, USA; drs00014@hsc.wvu.edu; 3 Department of Pharmacology and Toxicology, Michigan State University, East Lansing, MI 48824, USA; lauverda@msu.edu (D.A.L.); wattss@msu.edu (S.W.W.)

**Keywords:** perivascular adipose tissue, thoracic aorta, adipogenesis, hypertension

## Abstract

Cardiovascular diseases such as hypertension alter thoracic aorta structure. The role that the outer layer of the aorta, its perivascular adipose tissue (PVAT), plays in the pathogenesis of these alterations is poorly understood. In the descending thoracic aorta, PVAT is organized into three distinct strips: one located anterior to the aorta (AP) and two positioned laterally (LP). Genetic tracing indicates differences in the ontogeny of LP and AP, but the implications of these developmental differences and PVAT distribution on adipocyte development remain unknown. We hypothesize that the anatomical location of adipocyte progenitors influences their adipogenic potential and vasoactive functions. PVAT from LP and AP was collected from male SD rats at 10 wks of age (n = 7) to harvest adipocyte progenitors that were differentiated to adipocytes in adipogenic media. Adipogenesis was evaluated after induction and we performed next-generation RNA-seq on progenitors and adipocytes. We then employed Gene Set Enrichment Analysis for enrichment and network analyses. LP progenitors exhibited a 1.13-fold higher adipogenesis rate compared to those from AP. DEG analysis revealed LP had higher expression of adipogenic regulators and basal collagens *Col4a2* and *Col4a4*. When challenged with angiotensin-II, adipocyte progenitors from LP maintained their adipogenic capacity and adipocytes from the same site maintained their secretion of adiponectin at higher rates than AP cells. However, treatment with a Piezo1 mechanoreceptor agonist reduced LP’s adipogenic capacity and diminished their adiponectin secretion. These findings highlight site-specific differences in adipogenic activity, extracellular matrix composition, and the secretion of the vasoactive adipokine adiponectin between the LP and AP PVAT strips of the thoracic aorta, suggesting potential functional distinctions in vascular health and disease.

## 1. Introduction

During the pathogenesis of cardiovascular diseases such as hypertension and atherosclerosis, the descending thoracic aorta volume, wall thickness, and stiffness increase [1,2,3]. In hypertension, this remodeling process is well characterized in the aorta’s tunicas intima, media, and adventitia. However, changes occurring in the fourth layer, the perivascular adipose tissue (PVAT), during hypertension and other cardiovascular diseases are not well understood, due in part to the fragmented understanding of the biology of the tunica adiposa.

The descending thoracic aorta’s PVAT is distributed in three strips of tissue defined by anatomical landmarks: one strip is located anterior to the aorta (AP), while the other two (LP) are positioned laterally to the vessel and are adjacent to the thoracic vertebrae [4]. In both humans and rodents, the LP are larger, and exhibit dynamic populations of brown and white adipocytes that change during development, aging, and disease [5,6,7].

As in other adipose depots, adipocyte populations in the thoracic aorta PVAT are maintained by adipogenesis of adipocyte progenitors. These cells descend from fibroblast-like pluripotent stem cells. Genetic tracing in rodents indicates LP’s adipocytes descend from Sm22α^+^ and Myf5^+^ progenitors while AP’s are mostly from Sm22α^+^ origin [4]. The adipogenesis process prepares progenitors to exert functions typical of adipocytes including fatty acid and triglyceride synthesis, production and synthesis of adipokines, triglyceride hydrolysis, and export of fatty acids to be used as energy substrates by other tissues [8]. A unique aspect of PVAT is its vasoactive capacity. Adipocytes from this depot secrete adipokines such as adiponectin, which are potent vasorelaxants [9]. Therefore, maintaining a healthy and abundant population of adipocytes in PVAT may be important for cardiovascular health.

Currently, the implications of the unique anatomical distribution of the thoracic aorta PVAT (i.e., LP and AP) and its ontology on the development and function of its adipocytes are unknown. We hypothesize that the anatomical location of adipocyte progenitors influences their adipogenic potential and their vasoactive function. Here we demonstrate that AP and LP progenitors and adipocytes are not similar populations and that this diversity has implications on their biology as energy-storing cells and their vasoactive functions.

## 2. Materials and Methods

### 2.1. Animals

Male Sprague–Dawley rats (8–10 weeks old; Charles River Laboratories, Portage, MI, USA, RRID: SCR_003792) were housed in a temperature-controlled room (22 °C) under a 12:12 h light–dark cycle with environmental enrichment in standard cages. Rats had ad libitum access to standard chow and distilled water. All procedures were approved by the Michigan State University Institutional Animal Care and Use Committee (protocol no. PROTO202000009) and complied with the *Guide for the Care and Use of Laboratory Animals*, 8th edition [10]. Rats were anesthetized with an intraperitoneal injection of 60–80 mg/kg of pentobarbital. Deep anesthesia was verified by the lack of paw pinch and eye-blink reflexes, and death was assured by pneumothorax.

### 2.2. Adipocyte Progenitors Isolation and Culture

The thoracic aorta, along with its PVAT, was dissected and immersed in Krebs–Ringer Bicarbonate Buffer (KRBB, Teknova, Hollister, CA, USA, Cat N° H1030) supplemented with 100 U/mL penicillin, 100 µg/mL streptomycin, 0.25 µg/mL amphotericin B, and 50 µg/mL gentamicin. PVAT adipocyte progenitors were isolated as described [11]. The PVAT was separated from the thoracic aorta under a dissection stereoscope, and ~50 mg fragments were minced in 1–3 mm fragments and transferred into 24-well plates. After 5 min of incubation, 200 µL of MesenPro RS Complete Medium (Thermo Fisher Scientific, Waltham, MA, USA, Cat N° 12746012) was carefully added, and the tissue fragments were incubated at 37 °C. The medium was changed every 48 h until adipocyte progenitors migrated from within the tissue and reached confluency on the plate surface, at which point they were passed into flasks for expansion. Upon passage to flasks, cells were expanded as previously described [12].

### 2.3. Adipogenesis Experiments

Adipocyte progenitors were seeded in 6-well plates and induced to differentiate into adipocytes in standard adipogenic media alone, as previously described by our group [12]. Adipogenesis was assessed by staining neutral lipids with BODIPY 493/503 (Thermo Fisher, cat. no. D3922) and nuclei with NucSpot^®^ Live 650 (Biotium, Fremont, CA, USA, cat. no. 40082). Adipogenic activity was quantified as the ratio of BODIPY fluorescence intensity to nuclei count. Live-cell imaging was performed every 6 h over 4 days using the IncuCyte^®^ S3 system. Image analysis after 4 days in culture was conducted with IncuCyte ZOOM™ software (v2018A).

### 2.4. Angiotensin II and Yoda1 In Vitro Experiments

Adipocyte progenitors were plated in 96-well plates and induced to adipogenesis as previously described [12]. The cells were then exposed for 7 days to either the vasoactive peptide angiotensin II (1 µM; MedChem Express, Monmouth Junction, NJ, USA, Cat. No. HY-13948) or the Piezo1 mechanosensor agonist Yoda1 (10 µM; Tocris, Avonmouth, Bristol, UK, Cat. No. 5586), following protocols established by our group [12] and others [13]. After the 7-day treatment period, cells were harvested to assess triglyceride content using the Triglyceride-Glo kit (Promega, Madison, WI, USA, Cat. No. J3161) and adiponectin levels using the Rat Total Adiponectin/Acrp30 ELISA kit (R&D Systems, Minneapolis, MN, USA, Cat. No. RRP300), following manufacturers’ instructions.

### 2.5. RNA Isolation and Purification

RNA was isolated using the Maxwell^®^ RSC simplyRNA cells kit (AS1390, Promega) according to previously published protocols [12]. The purity, concentration, and integrity of mRNA were checked using a NanoDrop One^C^ spectrophotometer (Thermo Scientific, Wilmington, DE, USA) and an Agilent Bioanalyzer 2100 system (Agilent Technologies, Santa Clara, CA, USA). All samples had a 260:280 nm ratio between 1.9 and 2.1 and RNA integrity number ≥ 8.

### 2.6. RNA-Seq Analyses

RNA samples were sent to Novogene Corporation Inc. (Sacramento, CA, USA) for sequencing in the Illumina platform. Data quality control was performed with FastQC v.0.11 (www.bioinformatics.babraham.ac.uk/projects/fastqc/ accessed on 5 May 2024). After data filtering, clean reads were mapped to the Rnor_6.0 reference genome using HISAT 2.1.0 [14]. After genome mapping, featureCounts v1.22.2 was used to count the number of reads and quantify the number of transcripts (FpKM) to perform differential expression analysis ([15]).

Gene count matrix was filtered for genes with low transcription abundance, and features with constant values (either 0 or empty) were removed. The gene counts were then normalized using the DESEQ2.0 model followed by differential expression analysis [16]. Principal components analysis (PCA) and 3D PCA analyses were plotted with R v4.0.2 using the plotlib package v 4.10.1. Genes with significant thresholds and false discovery rates (FDRs) < 0.05 were defined as Differential Expressed Genes (DEGs) and captured for further analysis. All data is available in NCBI Gene Expression Omnibus (accession number: GSE244664).

### 2.7. Enrichment Analysis

Gene Set Enrichment Analysis (GSEA) was conducted utilizing the ClusterProfiler package v 4.5.2 in R (v4.0.2), aimed at identifying enriched pathways within the gene expression profiles derived from RNA-seq data sets [17]. Genes were ranked according to their average fold change and statistical significance, False Discovery Rate (FDR). Gene sets from the comprehensive Gene Ontology (GO) database and molecular signature database were used to perform the enrichment. Additionally, to assess whether collagens and integrin sets were enriched among the differentially expressed genes, we performed a hypergeometric test using the set of all detected transcripts as the background. Enrichment significance thresholds were determined using the Benjamini–Hochberg procedure, with a cutoff of FDR < 0.05, and Normalized Enrichment Scores were used to depict the prediction of activated and suppressed pathways.

### 2.8. Statistical Analysis

Data were analyzed by one- or two-way ANOVA using JMP (v 17, JMP Statistical Discovery, Cary, NC, USA) and GraphPad Software (v10, GraphPad, San Diego, CA, USA). Residuals of the model were checked for normal distribution—random effect of the rat within the treatment and mechanical stretch. Post hoc comparisons were performed using Tukey’s adjustments test. Statistical significance was set at *p* ≤ 0.05.

## 3. Results

### 3.1. AP and LP Adipocyte Progenitor Cells Exhibit Different Adipogenic Potential

**Figure 1:**
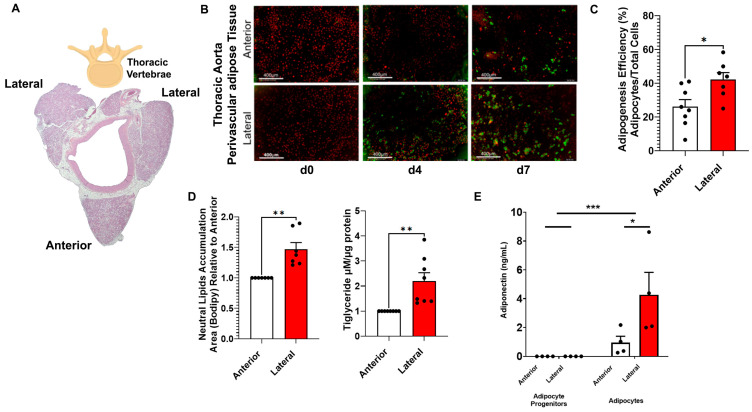
Adipogenesis and lipogenesis in adipocytes from anterior and lateral thoracic aortic perivascular adipose tissue (PVAT) strips. (**A**) Low magnification representative image of the rat’s thoracic aorta with its three strips of PVAT, one anterior and two lateral. (**B**) Representative images of adipocyte progenitors and adipocytes at 4 and 7 days post-induction (n = 7). Lipid droplets were stained with Bodipy (Thermo Fisher; green) and nuclei with NucRed (Biotium; red). Scale bar = 400 µm. (**C**) Adipogenic efficiency as calculated by the IncuCyte imager (Sartorius; number of cells with one or more lipid droplets over total number of cells per well); n = 7. (**D**) Neutral lipid accumulation and triglycerides content (using Triglyceride-Glo Assay) relative to Anterior after 7 d in culture; n = 7. (**E**) Adiponectin content in media and cells as measured by ELISA (Thermo Fisher) after 7 d in culture; n = 4. Dots indicate individual data points. Error bars indicate SEM, * *p* < 0.05, ** *p* < 0.01, *** *p* < 0.001.

Adipocyte progenitors were harvested from the thoracic aorta PVAT. Similar to what is observed in mice, the thoracic PVAT in the rat is distributed in three strips of tissue: one strip is located anterior to the aorta (AP), while the other two (LP) are positioned laterally to the vessel and are adjacent to the thoracic vertebrae (Figure 1A). To evaluate the adipogenic capacity of AP and LP, adipocyte progenitors were exposed to standard adipogenic media for 4 d. Adipocyte progenitors from LP exhibited a higher adipogenesis rate compared to those from AP (Figure 1B,C). Concurring with this observation, LP adipocyte progenitors cultured for 7 d had larger lipid droplets and higher triglyceride content compared to those from AP (Figure 1B,D). In addition, after 7 d of culture, adipocytes from LP secreted more adiponectin than those from AP. These results indicate that LP adipocyte progenitors have a higher adipogenic capacity than those of AP, and the resulting mature adipocytes accumulate more lipids (Figure 1D).

### 3.2. Thoracic Aorta AP and LP Adipocyte Progenitor Cells and Adipocytes Present Divergent Transcriptome Profiles

**Figure 2:**
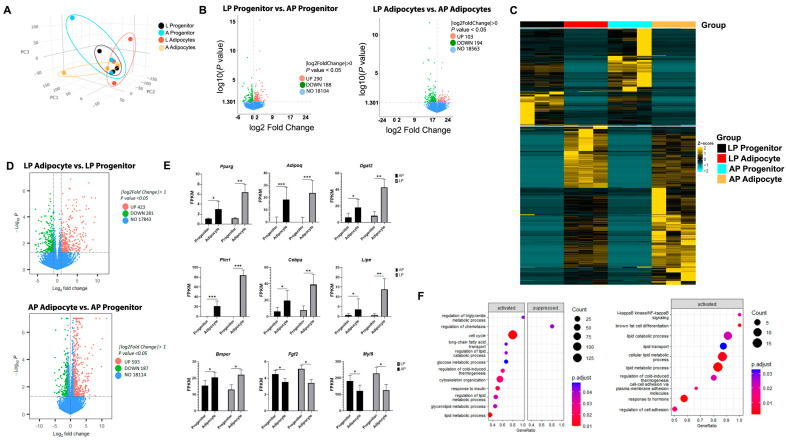
Adipocyte progenitors and adipocytes from the thoracic aorta’s anterior and lateral PVAT exhibit different transcriptomic profiles. Adipose tissue samples were collected from anterior (AP) and lateral (LP) PVAT of SD male rats (n = 3). (**A**). Principal component (3D) and clustering analyses show gene transcription comparisons between adipocyte progenitors and mature adipocytes in AP and LP. (**B**). Volcano plots of differentially expressed genes in adipocyte progenitors and adipocytes from LP when compared to AP. Overall results of FPKM cluster analysis from LP and AP adipocyte progenitors and adipocytes (using log_2_ (FPKM + 1) values). Red = high expression levels; green = low expression levels. Colors ranging from red to green indicate that log_2_ (FPKM + 1) values range from large to small. (**C**). Heatmap displays gene expression levels, with rows representing genes and columns representing samples (n = 3 in each group). Color intensity indicates z-score expression magnitude, from low (cyan) to high (yellow). (**D**). Volcano plots of differentially expressed genes in LP and AP adipocyte progenitors when compared to adipocytes from the same site. (**E**). Fragments per kilobase per million mapped reads (FPKM) of gene markers of adipogenesis (*Pparg*, *Adipoq*, *Dgat2*, *Plin1*, *Cebpa*, *Lipe*) and adipocyte (*Bmper*) and fibroblast (Fgf2, Myl9) phenotypes. Data are means ± SEM. Comparison lines with *, **, *** differ (*p* < 0.05, 0.01, and 0.001, respectively). (**F**). Dot plots visualization of GSEA results, pathways were sorted along the x-axis by Normalized Enrichment Score (NES) to indicate activation (positive NES) or suppression (negative NES). Dot size reflects the gene count in each pathway, while color denotes significance levels, facilitating quick identification of key regulated pathways.

Next, we collected AP and LP adipocyte progenitors before (0 d, progenitor) and after (7 d, adipocyte) inducing adipogenesis, and performed NGS RNA-seq. Three-dimensional PCA and clustering analyses show contrasting gene expression patterns between progenitors and adipocytes and between AP and LP at both stages (Figure 2A). DEGs showed that at the progenitor stage, AP cells had 290 upregulated and 188 downregulated genes compared to LP (Figure 2B). A similar pattern was observed at the adipocyte stage, where AP adipocytes had 103 genes upregulated and 194 genes downregulated when compared to LP adipocytes. The transcriptomic profiles of LP and AP adipocyte progenitors and adipocytes were represented in the heatmap of gene expression levels (Figure 2C).

### 3.3. Adipogenesis Activates Specific Gene Pathways in Thoracic Aorta AP and LP Adipocyte Progenitor Cells and Adipocytes

Adipogenesis activates specific gene networks in progenitor cells that initiate lipid accumulation processes and the synthesis and secretion of adipokines. AP adipocytes upregulated 593 and downregulated 187 genes when compared to AP progenitors (Figure 2D). In LP adipocytes, there was upregulation of 423 and downregulation of 281 genes (Figure 2D). Adipogenesis markers, including *Pparg*, *Adipoq*, *Dgat2*, *Plin1*, *Cebpa*, *Lipe*, and the marker of adipocyte commitment *Bmper*, were upregulated in adipocytes from both AP and LP compared to progenitors. In contrast, the expression of fibroblast phenotype markers *Fgf2* and *Myl9* decreased in adipocytes compared to progenitors independent of the site (Figure 2E). Gene set enrichment analysis (GSEA) identified pathways activated differentially in AP and LP. Processes related to triglyceride metabolic processes and fatty acid transport were activated in AP. In contrast, brown cell differentiation and cold-induced thermogenesis processes were more active in LP (Figure 2F).

### 3.4. Adipogenesis Alters Gene Expression of Collagens and Integrins in Thoracic Aorta AP and LP Adipocyte Progenitor Cells and Adipocytes

**Figure 3:**
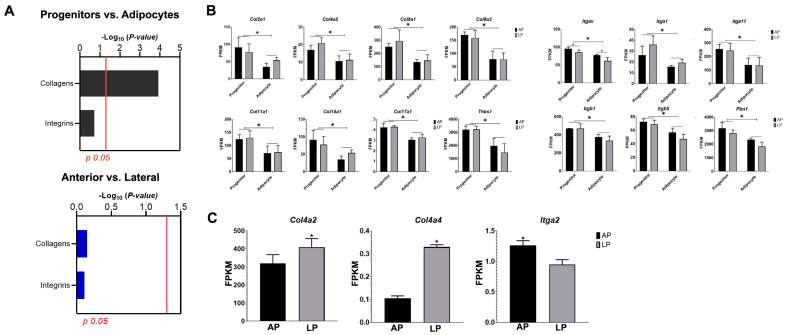
Adipogenesis Alters Extracellular Matrix Gene Transcription Patterns in Thoracic Aorta AP and LP Adipocyte Progenitor Cells. Adipose tissue samples were collected from anterior (AP) and lateral (LP) PVAT of SD male rats (n = 3). (**A**) Results of hypergeometric tests showing collagen and integrin gene family enrichment among differentially expressed genes in progenitors vs. adipocytes (top, gray) and anterior vs. lateral (bottom, blue) comparisons. The vertical red line indicates *p* = 0.05. Fragments per kilobase per million mapped reads (FPKM) of collagens and thrombospondin 1 and integrins and fibronectin 1 (**B**) detected in adipocyte progenitors from AP and LP and adipocytes after 7 days of adipogenesis induction. (**C**) Collagens and integrins were differentially expressed in both adipocyte progenitors and adipocytes from AP and LP. Data are means ± SEM. Comparison lines with * differ (*p* < 0.05).

Adipogenesis elicits changes in the gene transcription of extracellular matrix proteins in progenitors and adipocytes independent of their AP or LP origin (Figure 3A). Genes encoding for collagens *Col2a1*, *Col4a5*, *Col8a1*, *Col8a2*, *Col11a1*, *Col14a1*, *Col17a1*, and the glycoprotein thrombospondin 1 (Thbs1) were significantly enriched in our dataset compared to integrins (Figure 3B). Moreover, their expression levels declined after 7 days of adipogenesis induction (Figure 3B). Similarly, the integrins *Itga1*, *Itga11*, *Itgb1*, *Itgb5*, and *Itgav* and fibronectin (*Fbn1*) had higher expression in adipocyte progenitors vs. adipocytes independent of the site (Figure 3B). There was a site effect (AP vs. LP) on the expression of *Col4a2* and *Col4a4* that was higher in LP compared to AP without adipogenesis effects (Figure 3C). In contrast, the integrins *Itga2* and *Itgav* had higher transcription in the AP compared to LP (Figure 3B,C).

### 3.5. Thoracic Aorta AP and LP Adipocytes’ Responses to Angiotensin-II and Mechanosensor Stimulation Differ

**Figure 4:**
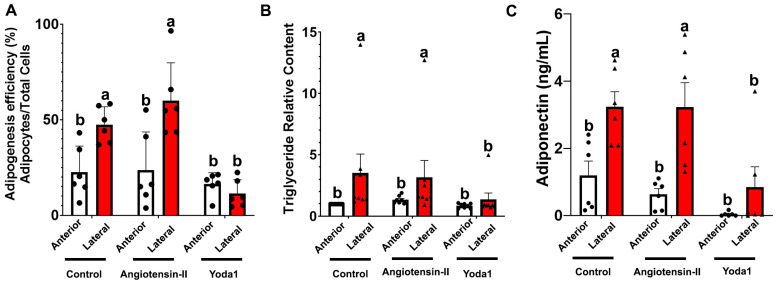
Adipogenesis in Thoracic Aorta AP and LP Adipocyte Progenitor Cells. Adipose tissue samples were collected from anterior (AP) and lateral (LP) PVAT of SD rats (n = 4). (**A**). Adipogenesis efficiency was examined using the IncuCyte imager (Sartorius; number of cells with one or more lipid droplets over total number of cells per well); n = 4. (**B**). Triglyceride content in cells were measured using Triglyceride-Glo Assay; n = 4. (**C**). Adiponectin secretion content from cells measured using ELISA (Thermo Fisher); n = 4. Dots in graphs are individual data points and error bars indicate the SEM. Bars with different superscripts ^a,b^ are different (*p* < 0.05).

During cardiovascular diseases such as hypertension, the PVAT of vessels like the thoracic aorta are subjected to stimulation by potent vasoconstrictors such as angiotensin-II and the activation of their mechanosensors like Piezo1 due to blood flow mechanical forces. Therefore, we exposed progenitors and adipocytes to angiotensin-II and the Piezo1 agonist yoda1. After 7 d of adipogenesis induction, we evaluated adipogenesis efficiency by determining the percentage of cells (nuclei count) with at least one lipid droplet (Bodipy stained). Adipocytes from LP and AP treated with control and angiotensin-II had higher adipogenesis efficiency than those treated with yoda1 (32.7, 36.5, and 13.9 ± 3.7%, respectively; *p* < 0.01). As expected, across all treatments, AP had lower adipogenesis efficiency than LP (18.7, 36.7 ± 3.0%, respectively; *p* < 0.01). AP cells had lower adipogenesis efficiency than LP when exposed to control (induction media) and angiotensin-II (Figure 4A). However, when cells were treated with yoda1, adipogenesis efficiency was drastically reduced in LP (Figure 4A). The enhanced adipogenesis was also reflected in higher triglyceride accumulation in control and angiotensin-II, compared to yoda1. Likewise, AP accumulated less triglycerides than LP which can be described as fold changes over the control AP values (Figure 4B).

Adiponectin expression was influenced by the adipogenesis efficiency and the treatment conditions. As adipogenesis was higher in LP compared to AP, adiponectin levels were correspondingly enhanced in LP across all treatments. Adipocytes treated with control and angiotensin-II exhibited higher adiponectin expression (ng/mL) than those treated with yoda1 (4.025, 5.212, and 1.256 ± 1.503, respectively; *p* < 0.05). Similarly, across all treatments, adiponectin expression (ng/mL) was significantly higher in LP than AP (2.623, 0.254 ± 0.752, respectively; *p* < 0.01). When exposed to control or angiotensin-II, AP cells had consistently lower adiponectin expression than LP (Figure 4C). Notably, treatment with yoda1 drastically reduced adiponectin expression in both AP and LP, with the effect being more pronounced in LP (Figure 4C).

## 4. Discussion

The aorta is one of the main conduit vessels, and structural changes in its four tunicas contribute to cardiovascular disease, including hypertension. While the intima, media, and adventitia tunicas have been well studied, the outermost layer, the tunica adiposa or PVAT, remains less understood. Here, we show that thoracic aorta PVAT in rats is arranged in two lateral (LP) and one anterior (AP) strip, and this anatomical distribution influences the adipogenic capacity of their adipocyte progenitor cells and their secretion of vasoactive factors like adiponectin.

### 4.1. Thoracic Aorta AP and LP Adipocytes’ Ontogeny Impact Their Function

Ruan and colleagues demonstrated distinct cellular lineages in murine thoracic aorta PVAT, where LP adipocytes originate from SM22α^+^ and My5^+^ progenitors while AP adipocytes arise solely from SM22α^+^ progenitors [4]. These lineages result in phenotypic differences: LP adipocytes exhibit a stronger brown adipocyte phenotype with higher UCP1 expression, whereas AP adipocytes resemble more typical white adipocytes. In this study, we show that rat descending thoracic aorta PVAT shares a similar anatomical distribution with discrete LP and AP strips. This distribution influences responses to adipogenic and vasomodulatory stimuli, with LP progenitors demonstrating greater adipogenic capacity than AP. One possible explanation is related to the adipogenic capacity of white adipocyte progenitors (e.g., those from AP) from visceral and thoracic white adipose depots. In vitro, ex vivo, and in vivo, these cells show a lower adipogenic capacity than those from white adipose tissue located in subcutaneous depots [18]. Another possible factor is the SM22α (transgelin) lineage of AP progenitors [4]. Transgelin is expressed by various cell types during development, including vascular smooth muscle cells, myocardial cells, and adipocyte progenitors [19]. Although typically associated with a vascular smooth muscle fate, transgelin expression is also essential for adipogenesis and osteoblast differentiation [20]. Notably, PPARγ knockout in transgelin-expressing cells in mice results in a PVAT-deficient phenotype [21].

Consistent with phenotypic differences, transcriptomic data from our RNA-seq analysis revealed distinct gene expression profiles in thoracic aorta PVAT strips at both the adipocyte progenitor and mature adipocyte stages. AP strip adipocytes, reflecting lower adipogenic potential, showed reduced expression of adipogenic markers and a more white-like transcriptomic profile, while LP adipocytes exhibited a more brown-like profile. This aligns with previous observations in murine aortic PVAT [4]. A similar pattern is seen in human periaortic fat: thoracic aortic PVAT near the vertebrae has a brown-like phenotype, whereas PVAT in the ascending and anterior thoracic aorta is more white-like, particularly in individuals with obesity [5] and older adults [22].

Results from the NGS RNA-seq analyses in the present study confirm significant transcriptomic changes associated with adipogenesis in cells from PVAT. These changes include activation of triglyceride synthesis pathways, alterations in cellular structure, and shifts in expression of genes encoding extracellular matrix proteins. Adipogenesis increased *Bmper* expression in both AP and LP adipocytes. *Bmper,* which encodes the bone morphogenic protein endothelial receptor, is a marker of adipocyte progenitors that preferentially differentiate into adipocytes in mice [23], rats [24], and humans [25]. Although its role in adipogenesis is not fully understood, *Bmper* appears necessary for initiating lipid accumulation via BMP4 signaling and enhancing insulin sensitivity [25]. As AP and LP adipocytes matured, *Bmper* expression increased, while fibroblast markers *Fgf2* and *Myl9* decreased. Sequencing data also showed that adipogenesis in AP and LP follows a transcriptomic pattern similar to other adipose tissue sites, with upregulation of key adipogenic genes such as *Pparg*, *Adipoq*, *Dgat2*, *Plin1*, *Cebpa*, and *Lipe*. Collectively, these findings indicate that adipogenesis in AP and LP strips resembles that in other adipose tissue sites. However, the transcriptional patterns in AP reflect its lower adipogenic capacity.

### 4.2. AP and LP Adipocytes in the Thoracic Aorta: Transcriptional Differences in ECM Genes

Adipogenesis involves more than just enabling adipocyte progenitors to store and mobilize lipids; it also remodels the ECM to provide a scaffold for newly formed adipocytes. Transcriptomic data from this study indicate minimal changes in the expression of fibril-forming collagens (*Col1*, *Col3*, *Col5*) and micro-fibrillar *Col6* in both AP and LP progenitors and adipocytes. This was expected, as the cultured adipocytes were not exposed to pro-fibrotic conditions, which are known to upregulate *Col1* and *Col5*, particularly in depots of myogenic origin [26]. Adipogenesis in both AP and LP also led to a reduction in the expression of cartilage-associated collagens Col2a1 and Col11a1, consistent with prior findings from our group, which showed *Col11a1* expression in progenitors but not in mature adipocytes [27]. These results suggest AP and LP progenitors can produce these collagen types, which are strongly associated with calcification foci in the aorta [26]. Of particular relevance to PVAT remodeling during cardiovascular disease is the observed expression of *Col8a1* and *Col8a2* and their downregulation during adipogenesis. This finding is consistent with the high expression of these collagens in adipocyte progenitors within the aortic PVAT of 10-week-old rats, as well as their near absence in mature adipocytes [27]. Furthermore, our results align with reports of high *Col8a1* expression in the adventitia of carotid arteries [28]. Given the role of Collagen VIII in neovascularization and its association with atherosclerosis [28], altered expression of *Col8a1* and *Col8a2* in progenitors could represent a mechanistic target in aortic disease.

Adipogenesis also altered the expression of extracellular matrix components, including downregulation of thrombospondin-1 (*Thbs1*), a matricellular glycoprotein that promotes adipocyte and fibroblast progenitor proliferation [29]. This reduction likely reflects the mitotic arrest during adipocyte differentiation. Elevated *Thbs1* levels have been linked to adipose tissue inflammation in obesity and high-fat diet models [30,31], as well as increased blood pressure in hypertensive rats [32]. Our findings suggest that PVAT adipocyte progenitors may be a source of thrombospondin-1, and changes in its expression within these cells or other PVAT components could potentially trigger responses similar to those observed in obesity.

Integrins such as *Itgav* and *Itga11* were downregulated during adipogenesis, consistent with findings by Morandi et al. [33], who reported similar decreases in human adipose tissue stem cells. In their study, *Itgav* knockdown reduced the proliferation of adipocyte progenitors. Our snRNA-seq data also showed higher expression of these integrins in progenitors versus mature adipocytes in 10-week-old rats [27]. Therefore, reduced expression of *Itgav* and the collagen receptor *Iga11* may reflect mitotic arrest and activation of adipogenic pathways during PVAT adipocyte differentiation. A similar downregulation was observed for beta integrins *Itgb1* and *Itgb5*, and *Fibronectin 1* (*Fbn1*). These ECM proteins support the self-renewal of human pluripotent stem cells [34] and adipocyte progenitors in rodents [35]. While their activity is necessary for maintaining adipocyte populations, excessive activation is associated with the development of AT fibrosis.

To our knowledge, this is the first study to examine ECM transcription profiles in cells from AP and LP strips of the thoracic aorta PVAT. We found higher expression of *Col4a2* and *Col4a4* in LP adipocyte progenitors and adipocytes. These type IV collagens are located in the basement membrane separating endothelial cells from adipocytes [36]. Previous studies from our group demonstrated expression of both *Col4A2* and *Col4a4* in aortic PVAT adipocyte progenitors and adipocytes [27]. It remains unclear whether the higher expression of these collagens promotes adipogenesis and angiogenesis in LP compared to AP. Another ECM component showing site-specific differences was *Itga2*, which was expressed at lower levels in LP compared to AP. Lower expression in LP may be linked with the higher adipogenic capacity of progenitors from this site, as this integrin has been shown to inhibit the differentiation of human adipose-derived stem cells into adipocytes in a Rac-1-dependent manner [37]. Rac-1 activation induces changes in cellular morphology, enabling cells to accumulate lipids [37].

### 4.3. Thoracic Aorta AP and LP Adipocytes’ Anatomical Location Influences Responses to Vasomodulators

To our knowledge, this is the first report on differences in adiponectin secretion between distinct strips of aortic PVAT. We show that LP adipocytes secrete more adiponectin than AP adipocytes, and this response was modulated by Piezo1 activation and angiotensin-II. Given adiponectin’s well-established vasoactive roles, such as enhancing nitric oxide and hydrogen sulfide production and activating potassium channels, these findings may have implications for aortic lesion development and vascular responses in hypertension. Future studies should explore how regional differences in adiponectin secretion affect vasorelaxation and contribute to the greater incidence of vascular lesions in specific areas of the thoracic aorta.

Considering the limitations of the reductionistic approach used in this study, which included only adipocyte progenitors from male rats, future research should explore the effects of sex, interactions with other PVAT cell types—including immune cells—and the impact of the absence of LP or AP strips in in vivo experiments. A second limitation is that the adipogenic medium used in this study was specifically formulated for culturing adipocyte progenitors from thoracic PVAT [38]. It is possible that media optimized for enhancing differentiation in adipocyte progenitors from other depots may elicit different responses in cells from AP, given its white adipocyte profile. Regardless, this response to the medium highlights the phenotypical differences between AP and LP.

## 5. Conclusions

Results from this study confirm that, in rats, the descending thoracic aorta PVAT is organized into anterior (AP) and lateral (LP) strips, and this anatomical distribution influences the adipogenic potential of their adipocyte progenitor cells. LP adipocyte progenitors exhibited a higher adipogenesis rate than AP progenitors, along with increased expression of adipogenic regulators and collagens *Col4a2* and *Col4a4*. Anatomical location also affected adipocyte responses to Piezo1 activation and angiotensin-II, with LP cells maintaining their adipogenic capacity and secreting more adiponectin. Taken together, our findings highlight site-specific variations in adipogenesis, extracellular matrix composition, and vasoactive function, which may impact vascular health and disease risk. Future research should evaluate the impact of these biological differences on the vascular and metabolic function of thoracic aorta PVAT in vivo. Additionally, studies should assess how the unique anatomical characteristics of the LP region (e.g., its proximity and attachment to the spine and thoracic bone structures) influence the mechanical properties of the aorta and its associated structures and how these factors affect the vascular function of this large conduit vessel.

## Data Availability

Bulk RNA-seq data is available in NCBI Gene Expression Omnibus (accession number: GSE244664).
